# Common Driver Mutations in AML: Biological Impact, Clinical Considerations, and Treatment Strategies

**DOI:** 10.3390/cells13161392

**Published:** 2024-08-21

**Authors:** Tiffany Nong, Shefali Mehra, Justin Taylor

**Affiliations:** Sylvester Comprehensive Cancer Center, Miller School of Medicine, University of Miami, Miami, FL 33136, USA

**Keywords:** AML, driver mutations, *FLT3*, *NPM1*, *IDH*, *TP53*

## Abstract

Next-generation sequencing of samples from patients with acute myeloid leukemia (AML) has revealed several driver gene mutations in adult AML. However, unlike other cancers, AML is defined by relatively few mutations per patient, with a median of 4–5 depending on subtype. In this review, we will discuss the most common driver genes found in patients with AML and focus on the most clinically relevant ones that impact treatment strategies. The most common driver gene mutations in AML occur in *NPM1* and *FLT3*, accounting for ~30% each. There are now targeted therapies being tested or already approved for these driver genes. Menin inhibitors, a novel targeted therapy that blocks the function of the menin protein, are in clinical trials for *NPM1* driver gene mutant AML after relapse. A number of FLT3 inhibitors are now approved for *FLT3* driver gene mutant AML in combination with chemotherapy in the frontline and also as single agent in relapse. Although mutations in *IDH1/2* and *TP53* only occur in around 10–20% of patients with AML each, they can affect the treatment strategy due to their association with prognosis and availability of targeted agents. While the impact of other driver gene mutations in AML is recognized, there is a lack of data on the actionable impact of those mutations.

## 1. Introduction to Acute Myeloid Leukemia (AML): Heterogeneity, Molecular Insights, and the Role for Targeted Therapies and Genetic Profiling

Acute myeloid leukemia (AML) is a heterogeneous, hematologic malignancy that disrupts hematopoiesis in the bone marrow due to the clonal proliferation of immature myeloid cells [1,2,3]. Hematopoiesis is the process of stem cell differentiation and proliferation into functional components of peripheral blood and bone marrow [4]. AML is the most common form of leukemia, with the incidence increasing by 15% in the past three decades [5]. It is an aggressive, fatal disease, being the 11th most common cause of cancer deaths [6]. Cancer is a disease in the aging population, with elderly adults aged 60 to 75 with AML having unfavorable genetic profiles and mutations associated with poor prognosis [2,7]. In contrast, younger patients with AML typically have more favorable mutation profiles [8]. However, the development of next-generation sequencing has unraveled the molecular profile of AML for the investigation of novel targeted therapies, leading to curative potential in some patients [2,9].

**Figure 1:**
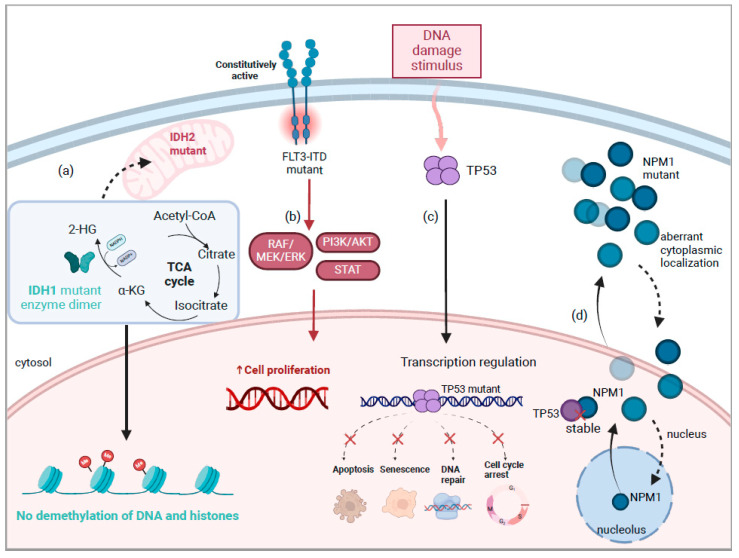
Key Driver Mutations and Cellular Pathways in AML Pathogenesis. (**a**) Mutant IDH1 (cytoplasm) and IDH2 (mitochondria) enzymes produce elevated levels of the oncometabolite 2-hydroxyglutarate (2-HG), which inhibits chromatin-modifying enzymes such as histone and DNA demethylases, leading to a block in cellular differentiation, contributing to oncogenesis. (**b**) *FLT3*-ITD mutations lead to constitutive activation of the FLT3 receptor, triggering downstream signaling pathways including RAF/MEK/ERK, PI3K/AKT, and STAT pathways. This continuous signaling promotes cell proliferation, survival, and differentiation blockade, contributing to leukemogenesis. (**c**) *TP53* mutations in AML impair the tumor suppressor functions of p53, which is activated in response to DNA damage stimuli, leading to disrupted apoptosis, senescence, DNA repair, and cell cycle arrest. This results in unchecked cell proliferation, genomic instability, and enhanced leukemogenesis. (**d**) In AML with a mutated *NPM1* gene, *NPM1* is aberrantly expressed in the cytoplasm due to increased nuclear export (solid arrows) surpassing nuclear import (dotted arrow). Mutant *NPM1* fails to stabilize *TP53* in the nucleoplasm, which normally modulates stress response and growth suppression.

The pathogenesis of AML is driven by genetic alterations in cells that can affect signal transduction, epigenetic modification, DNA repair, and apoptosis (Figure 1) [1]. Due to its heterogeneity, genomic characterization is necessary to assess risk classification and monitor disease progression, residual disease, and therapeutic resistance. The integration of genetic profiling allows for personalized medicine and influences clinical decision-making, ultimately impacting patient outcomes [10]. Targeted therapies and small molecule inhibitors have been novel agents investigated to target genetic changes such as mutations in leukemic cells and offer a less toxic treatment option. The European Leukemia Net (ELN) 2022 recommendations have been widely adopted by clinicians and researchers as the most up-to-date guidelines for risk stratification and prognostic management of AML [11]. These guidelines consider the mutations mentioned in this review and classify patients with these as favorable, intermediate, and adverse risks.

The objective of this review is to introduce the most common driver mutations from each risk classification. We will discuss the biological consequences, clinical impact, and clinical trial data of novel treatment options for *FLT3*-ITD, *NPM1*, *TP53*, and isocitrate dehydrogenase 1 and 2 (*IDH1/2*).

## 2. *NPM1* Mutations in AML: Genetic Landscape, Clinical Features, and Therapeutic Innovations

### 2.1. The Role of NPM1 Mutations in Leukemogenesis Synergistic Genetic Interactions: Impact of Co-Occurring FLT3-ITD Mutations in AML

*NPM1* mutations are among the most frequent genetic alterations in AML, occurring in approximately 25–30% of adult AML cases [12,13]. These mutations typically involve small insertions in exon 12, resulting in a frameshift that creates a novel C-terminus [14]. This alteration disrupts the nuclear localization signals (NLS) of the NPM1 protein, leading to its aberrant accumulation in the cytoplasm [15] (Figure 1d). Normally, NPM1 shuttles between the nucleus and cytoplasm, playing a crucial role in ribosome biogenesis, chromatin remodeling, and genomic stability. The cytoplasmic localization of mutant NPM1 disrupts its interactions with alternate reading frame (ARF) protein, which is crucial for stabilizing p53 by inhibiting MDM2-mediated degradation [16]. As a result, the mutant NPM1 protein impairs the p53 tumor suppressor pathway, reducing p53-mediated apoptosis and allowing myeloid cells to survive and proliferate unchecked [17]. Additionally, the mutant NPM1 protein disrupts the nuclear export of ribosomal proteins and other tumor suppressors, including ARF, further enhancing cellular proliferation and survival [18]. This mislocalization also impairs the assembly and function of the nucleolus, leading to defects in ribosomal RNA processing and biogenesis. These disruptions in nucleolar function contribute to genomic instability and the leukemogenic process [14,15].

### 2.2. Synergistic Genetic Interactions and Prognostic Significance: Impact of Co-Occurring FLT3-ITD Mutations in AML

*NPM1* mutations often co-occur with other genetic alterations, notably *FLT3* internal tandem duplications (*FLT3*-ITD), which are found in 30–40% of *NPM1*-mutated AML cases [19,20]. The coexistence of *FLT3*-ITD mutations exacerbates the leukemic phenotype through enhanced activation of signaling pathways involved in cell proliferation and survival, such as the PI3K/AKT and RAS/MAPK pathways [21,22]. This synergistic interaction between *NPM1* mutations and *FLT3*-ITD significantly impacts the pathogenesis and clinical behavior of AML [23]. Studies have demonstrated that patients with concurrent *NPM1* and *FLT3*-ITD mutations exhibit a more aggressive disease course, characterized by higher leukocyte counts and a higher incidence of extramedullary involvement. This genotype–phenotype correlation underscores the critical role of these mutations in driving AML pathogenesis and highlights the complexity of genetic interactions in this hematologic malignancy [13,17,24].

Moreover, *NPM1* mutations in AML are often associated with a favorable prognosis, particularly without concurrent high-risk mutations such as *FLT3*-ITD. Studies have shown that patients with *NPM1*-mutated AML tend to have higher complete remission rates and longer overall survival compared to those without these mutations [14]. The favorable prognosis is primarily observed in patients without *FLT3*-ITD or other adverse cytogenetic abnormalities. For instance, patients with *NPM1* mutations and wild-type *FLT3* exhibit a 5-year overall survival rate of approximately 60–70%, significantly better than patients with other AML subtypes [13]. Despite the generally positive outlook for *NPM1*-mutated AML, the presence of co-occurring mutations can alter this prognosis. For example, the co-occurrence of *FLT3*-ITD mutations often mitigates the favorable prognosis conferred by *NPM1* mutations, resulting in a more aggressive disease course and poorer outcomes [25]. In a prospective study of 1540 patients, it was found that the prognosis of AML was significantly influenced by the presence or absence of other driver mutations, rather than by *NPM1* mutations alone [17]. This underscores the importance of considering the mutational landscape as a whole when assessing prognosis in AML. *NPM1* mutations are also notable for their impact on response to chemotherapy. Patients with *NPM1*-mutated AML often show increased sensitivity to induction chemotherapy, resulting in higher rates of complete remission [14]. However, the presence of additional mutations such as *FLT3*-ITD can lead to shorter durations of remission and higher relapse rates. Thus, while *NPM1* mutations are a key prognostic biomarker in AML, clinical outcomes are heavily influenced by the broader genetic context in which they occur [26].

### 2.3. Conventional Chemotherapy and Innovations in Targeted Therapy: Current Treatment Approaches for NPM1-Mutated AML, Efficacy of Menin Inhibitors and Combination Strategies

Despite the clear genetic and prognostic implications of *NPM1* mutations in AML, there are currently no FDA-approved targeted therapies specifically addressing this mutation [13]. As such, the mainstay of treatment for *NPM1*-mutated AML remains conventional chemotherapy, often comprising an anthracycline and cytarabine-based regimen [27]. These patients typically achieve high complete remission rates, ranging from 70% to 90%, particularly in the absence of *FLT3*-ITD mutations. Supportive care, including hematopoietic growth factors, transfusions, and prophylactic antibiotics, plays a crucial role in managing treatment-related toxicities and complications [28]. Moreover, allogeneic hematopoietic stem cell transplantation (HSCT) is considered in cases of relapsed or refractory disease, although its role in *NPM1*-mutated AML is still being delineated [29].

Emerging therapies targeting the *NPM1* mutation in AML have shown promise in preclinical and early clinical studies, offering hope for improved outcomes in this subset of patients. Menin is a protein encoded by the multiple endocrine neoplasia 1 (MEN1) gene and was originally known as a tumor suppressor protein in endocrine glands. However, now it has been discovered to play a role in leukemogenesis. It is a nuclear protein expressed in multiple tissues that acts as a scaffold protein, interacting with chromatin regulators and transcription factors to regulate gene expression [30]. Menin inhibitors are small molecule inhibitors that have been discovered to block the interactions between menin and other proteins [31]. One such agent, revumenib (SNDX-5613), a menin inhibitor, was the first to disrupt the interaction between menin and MLL1, a critical component in the leukemogenesis driven by *NPM1* mutations [32]. Preclinical studies demonstrated that revumenib effectively inhibits the proliferation of *NPM1*-mutant leukemic cells by reactivating differentiation pathways and inducing apoptosis. A phase I/II clinical trial (NCT04065399) has shown promising results, with an ORR of 53% and a CR rate of 33% in patients with *NPM1*-mutant AML [32,33].

Another promising menin inhibitor, Ziftomenib (KO-539), is also being evaluated for its efficacy in targeting *NPM1* mutations. Preclinical studies have demonstrated that Ziftomenib effectively inhibits the proliferation of *NPM1*-mutant leukemic cells by reactivating differentiation pathways and inducing apoptosis [34]. A phase I/II clinical trial (NCT04067336) is currently evaluating the safety and efficacy of Ziftomenib in patients with relapsed or refractory AML, including those with *NPM1* mutations [35]. Early results from this trial have shown promising response rates, with an ORR of 29% in the *NPM1*-mutant cohort [34].

Additionally, DSP-5336 is a menin inhibitor currently being evaluated for its potential to target *NPM1*-mutant AML. Preclinical studies suggest that DSP-5336, similarly to other menin inhibitors, disrupts the menin–MLL1 interaction, leading to the inhibition of leukemic cell proliferation and induction of differentiation and apoptosis. Specifically, DSP-5336 has been shown to decrease the expression of downstream target genes such as HOXA9 and MEIS1, which are crucial for the maintenance and proliferation of leukemic stem cells. This disruption results in the reactivation of differentiation pathways and the induction of cell cycle arrest, ultimately promoting apoptosis in *NPM1*-mutant leukemic cells. Furthermore, DSP-5336 has demonstrated potent antileukemic activity in various in vivo models, reducing tumor burden and extending survival in treated mice [36]. A phase I/II clinical trial (NCT04988555) is ongoing to evaluate its safety and efficacy in patients with relapsed or refractory AML [37].

Another emerging strategy involves the combination of menin inhibitors with other targeted therapies. For instance, preclinical studies have suggested that combining menin inhibitors with *IDH* inhibitors or BCL-2 inhibitors like venetoclax can enhance therapeutic efficacy by targeting multiple leukemogenic pathways simultaneously [17]. A phase II trial (NCT04493138) is investigating the combination of Ziftomenib with venetoclax and azacitidine in patients with newly diagnosed or relapsed/refractory AML harboring *NPM1* mutations. Preliminary data indicate that this combination is well-tolerated and has shown encouraging activity, with a complete remission (CR) rate of 45% [38].

In addition to menin inhibitors, other novel agents are being explored for their potential to target *NPM1*-mutant AML [12]. For example, inhibitors of the bromodomain and extraterminal (BET) proteins, which are involved in regulating gene expression, have shown activity in preclinical models of *NPM1*-mutant AML. These inhibitors work by downregulating the expression of genes that are critical for the survival and proliferation of leukemic cells. A phase I trial (NCT02419417) is currently evaluating the BET inhibitor CPI-0610 in combination with standard chemotherapy in patients with *NPM1*-mutant AML [39].

## 3. *FLT3* Mutations in AML: Pathogenesis, Clinical Impact, and Therapeutic Advances

### 3.1. Prevalence, Mechanism of Action, and Clinical Implications of Constitutive Receptor Activation with FLT3 Mutations

With a frequent mutation rate in AML of about 20–30% occurring with an intermediate prognosis and high relapse rates, the *FMS-like tyrosine kinase* 3 (*FLT3*) gene became a popular target within the past decade [3,7]. The *FLT3* internal tandem duplication (*FLT3*-ITD) mutation is the most common *FLT3* mutation and has been associated with greater inferior survival than the *FLT3* tyrosine kinase domain (*FLT3*-TKD) mutation, making *FLT3*-ITD more clinically relevant [3,20,40,41]. AML patients with *FLT3* mutations tend to have shorter periods of remission, more frequent relapses, and worse overall survival outcomes than AML patients without these mutations [42]. Normally, an FLT3 receptor binds to a ligand produced by cells in the hematopoietic environment, leading to receptor dimerization and autophosphorylation. This activates signaling pathways downstream like RAF/MEK/ERK, PI3K/AKT, and STAT to control the differentiation and growth of hematopoietic cells while inhibiting apoptosis (Figure 1b) [7]. In the *FLT3*-ITD mutation, the FLT3-ITD receptor dimerizes with the wild-type receptor. This leads to constitutive activation independently of binding to a ligand, leading to the potential for uncontrolled malignant proliferation of hematopoietic cells [4]. This constitutive activation is the reason for *FLT3*-mutated AML patients presenting with high blast counts at diagnosis. During clonal shifts, *FLT3* mutations are poorly conserved, meaning the sequence can vary significantly in clones of cancer cells over time.

### 3.2. The Complex Prognostic Landscape of FLT3-ITD Mutations in AML: Clonal Expansion, Relapse Risk, and the Impact of Co-Occurring Mutations

The process of clonal expansion in relapsed AML with *FLT3*-ITD mutation is associated with a high allelic ratio, indicating that a subclone at diagnosis has a growth advantage and becomes the dominant clone at relapse [43]. However, some studies show that allelic ratio does not necessarily contribute to the risk of relapse and prognosis, and it is no longer considered on the ELN 2022 risk classification. This was due to the lack of standardization in the assay to measure the *FLT3* allelic ratio [44,45]. Approximately 75% of AML patients continue to have the *FLT3*-ITD mutations at relapse from diagnosis, which indicates its role as a driver mutation [46]. Using *FLT3* mutations as the only biomarker for MRD has been discouraged due to false negative MRD relapses due to missed clonal shifts [47]. *FLT3*-ITD is associated with mutations in *DNMT3A*, contributing to an even worse prognosis. In contrast, it is also associated with mutations in *NPM1*, which has a favorable prognosis on its own, but the ELN 2022 guidelines now place *FLT3*-ITD mutations in the intermediate risk category regardless of *NPM1* co-occurrence [44]. *FLT3*-ITD is an important biomarker for prognosis; however, clinical outcomes are ultimately impacted by co-occurring mutations and characteristics at diagnosis.

### 3.3. Targeted FLT3 Inhibitors in AML: Clinical Efficacy and Evolution of Treatment Landscape with Midostaurin, Gilteritinib, Quizartinib, and Emerging Role of Crenolanib

Due to the poor outcomes of patients with *FLT3*-ITD mutations, the need for a targeted therapy became clear. *FLT3* inhibitors prevent downstream phosphorylation by competitively inhibiting ATP binding at the site of the tyrosine kinase domain [48]. The first *FLT3* inhibitor to be FDA approved in 2017 was midostaurin, which evolved the treatment landscape by being the first targeted therapy for AML to improve overall survival and is now approved as a first-line treatment in addition to standard chemotherapy in those with either an *FLT3*-TKD or *FLT3*-ITD mutation [49]. The phase III RATIFY study (NCT00651261) compared midostaurin + standard chemotherapy vs. placebo + standard chemotherapy in 717 patients. It significantly improved median overall survival (74.7 months vs. 25.6 months), with the 4-year overall survival rate being 51.4% vs. 44.3%, respectively [48]. Next, in 2018, came the approval of gilteritinib for relapsed/refractory AML patients, which targets both *FLT3*-ITD and *FLT3*-TKD mutations. It was developed to target relapsed/refractory AML due to the inability of midostaurin to establish a durable remission. This phase III trial (NCT02421939) compared the use of gilteritinib vs. salvage chemotherapy and respectively found a longer median overall survival (9.3 vs. 5.6 months) and higher complete remission with full or partial hematologic recovery (34.0% vs. 15.3%) [50]. Due to the mechanisms of resistance that still presented with gilteritinib over time, quizartinib was developed to escape resistance and is distinguished from other *FLT3* inhibitors by binding to the hydrophobic region instead of the active site of *FLT3* kinase and while it is in its inactive conformation [51]. Quizartinib was FDA-approved in 2023 following a phase III QuANTUM trial (NCT02039726) that compared the use of quizartinib vs. salvage chemotherapy in AML patients with *FLT3*-ITD mutations only and respectively led to a longer median overall survival in the intent-to-treat population (6.2 vs. 4.7 months) and when censored for stem cell transplant (5.7 vs. 4.6 months) [52]. It included older patients up to age 75, as compared to the RATIFY study in midostaurin, illustrating the feasibility of using quizartinib in older populations that tend to have more toxicities. It is now approved for use as a first-line therapy in newly diagnosed AML with *FLT3*-ITD mutation. An emerging therapy that received FDA fast-track designation in 2017 is crenolanib, which has shown promising results in relapsed/refractory AML [53]. It has the potential to overcome mechanisms of resistance that are present in the use of quizartinib, such as *FLT3* mutations at D835 [54]. A phase III trial (NCT03258931) comparing the efficacy of midostaurin vs. crenolanib is currently underway. Another *FLT3* inhibitor being investigated in the early trial phase is FF-10101, which can escape mechanisms of resistance by having activity against *FLT3*-ITD and *FLT3*-TKD, as well as extracellular activating mutations [55].

## 4. *TP53* Mutations in AML: Pathogenesis, Clinical Impact, and Emerging Therapeutic Strategies

### 4.1. Prevalence, Mechanistic Disruption of Cell Function with TP53 Mutations in AML

*TP53* mutations represent a significant subset of genetic alterations in AML, with a prevalence of approximately 5–10% in newly diagnosed cases [25,56]. The *TP53* gene, located on chromosome 17p13.1, encodes the tumor protein p53, which is a crucial regulator of genomic stability [15,57]. In AML, *TP53* mutations often result in missense mutations that produce a defective p53 protein, which accumulates abnormally within cells [58]. Normally, p53 acts as a transcription factor that regulates the expression of genes involved in the DNA damage response, cell cycle arrest, and apoptosis (Figure 1c) [17,59]. Upon detecting DNA damage, p53 induces the transcription of genes such as p21, GADD45, and PUMA, which mediate cell cycle arrest and apoptosis to prevent the propagation of damaged cells. However, mutant p53 proteins lose this functionality, impairing the DNA damage response and allowing cells with genetic instability to survive and proliferate unchecked [25,60].

The defective p53 protein is unable to bind to DNA effectively, which prevents it from activating the transcription of its target genes essential for DNA repair and apoptosis. This loss of function results in the failure to arrest the cell cycle in response to DNA damage, leading to the accumulation of additional genetic abnormalities [56]. Furthermore, the inability of mutant p53 to induce apoptosis means that damaged cells can continue to proliferate, contributing to leukemogenesis. Additionally, mutant p53 proteins can exert a dominant-negative effect by forming hetero-oligomers with wild-type p53, thereby inhibiting its tumor suppressor functions.

Mutant p53 can also gain oncogenic functions, known as gain-of-function (GOF) mutations, which further drive tumorigenesis [61]. These GOF mutations can alter the interaction of p53 with other cellular proteins, such as transcription factors and components of the chromatin remodeling machinery, disrupting normal cellular processes. For example, mutant p53 can interact with the transcriptional coactivator p300/CBP, leading to aberrant gene expression that promotes cell survival and proliferation [62]. Additionally, the altered interactions of mutant p53 with proteins involved in DNA repair, such as BRCA1 and RAD51, can further compromise genomic stability [58,63,64].

Overall, *TP53* mutations in AML result in a defective p53 protein that fails to maintain genomic stability, regulate the cell cycle, and induce apoptosis. This loss of function, combined with potential gain-of-function effects, drives leukemogenesis by allowing the survival and proliferation of genetically unstable cells, contributing to the complexity and aggressiveness of the disease [60].

### 4.2. Prognostic Implications of TP53 Mutations in AML: Challenges with Treatment Resistance and Poor Clinical Outcomes

*TP53* mutations in AML are associated with a markedly poor prognosis, characterized by low complete remission rates, high relapse rates, and poor overall survival [25,56]. In these patients, the presence of *TP53* mutations contributes to genomic instability, leading to a more aggressive disease phenotype and resistance to conventional therapies [65,66]. The prognostic significance of *TP53* mutations is highlighted by their association with poor treatment outcomes. For instance, patients with *TP53*-mutant AML typically exhibit a median overall survival of less than 6 months, significantly shorter than those with wild-type *TP53* [17,67].

Biallelic *TP53* mutations are strongly associated with extremely poor outcomes; in contrast, monoallelic *TP53* mutations, while still adverse, are generally associated with a slightly better prognosis, with a 2-year OS of 4% versus 43%, respectively [68]. This poor prognosis persists despite intensive chemotherapy or hypomethylating agents, as *TP53*-mutant leukemic cells often exhibit resistance to these treatments [13]. Moreover, *TP53* mutations are frequently observed in therapy-related AML and secondary AML, both of which are associated with particularly poor clinical outcomes [56,66]. The co-occurrence of *TP53* mutations with other high-risk genetic alterations, such as complex karyotypes, further exacerbates the prognosis. In a study of 944 patients, it was found that *TP53* mutations were correlated with specific copy number alterations and a monosomal karyotype, both indicators of dismal outcomes [25,65].

### 4.3. Novel Therapeutic Approaches: Potential of APR-246, MDM2 Inhibitors, and Combination Therapies

Emerging therapies targeting *TP53* mutations in AML are of significant interest due to the poor prognosis associated with this genetic alteration. One of the most promising agents in this context is APR-246 (eprenetapopt), which aims to restore the normal function of mutant p53 by refolding the defective protein into its wild-type conformation [69]. In preclinical models, APR-246 has demonstrated the ability to induce apoptosis and inhibit tumor growth in *TP53*-mutant leukemic cells [70]. A phase II clinical trial (NCT03745716) evaluating APR-246 in combination with azacitidine in patients with *TP53*-mutant myelodysplastic syndromes (MDS) and AML reported a complete remission (CR) rate of 50% and a median overall survival (OS) of 10.8 months. However, it is important to note that despite initial promising results, subsequent studies have not confirmed long-term efficacy, leading to the discontinuation of some trials involving APR-246 in combination with azacitidine [71].

Another novel approach involves targeting MDM2, a negative regulator of p53 that is often overexpressed in cancers, including AML. MDM2 inhibitors, such as idasanutlin, work by reactivating the p53 pathway, leading to apoptosis in p53-wildtype and potentially in *TP53*-mutant cells through synthetic lethality mechanisms [34]. A phase I/II trial (NCT02545283) investigating idasanutlin in combination with cytarabine in relapsed/refractory AML showed an ORR of 29%, with some patients achieving durable remissions [72]. However, these results have not been sufficient to advance the treatment into later-stage clinical trials, highlighting the challenges in targeting the *TP53*-mutant AML [72].

Combination therapies are also being explored to enhance the efficacy of emerging agents. For example, a phase Ib/II study (NCT03931291) is evaluating the combination of APR-246 with venetoclax and azacitidine in *TP53*-mutant AML [73]. Preliminary results have shown that this triplet combination is well-tolerated and has led to high response rates, with complete remission and an incomplete hematologic recovery rate of 58% in the evaluable population. This combination aims to exploit the synergistic effects of reactivating p53, inducing apoptosis through BCL-2 inhibition, and promoting differentiation with azacitidine [71].

Furthermore, immune checkpoint inhibitors are being investigated in *TP53*-mutant AML due to the potential for enhanced immune evasion in these tumors. A phase II trial (NCT04284787) is assessing the combination of the anti-PD-1 antibody pembrolizumab with azacitidine in *TP53*-mutant AML and MDS [74]. Early data suggest that this combination can induce immune responses and lead to clinical remissions, highlighting the potential for immunotherapy in this difficult-to-treat population [74]. Nonetheless, the long-term efficacy and safety of immune checkpoint inhibitors in this context remain under investigation, with mixed results from various trials.

## 5. *IDH1/2* Mutations in AML: Pathogenesis, Clinical Impact, and Therapeutic Advances

### 5.1. Metabolic Disruption and Epigenetic Alterations: The Role of IDH Mutations in Leukemogenesis

*Isocitrate dehydrogenase* (*IDH*) exists as two isoforms: IDH1 localized in the cytosol, and IDH2 localized to the mitochondria, and they are mutated in about 20% of AML. Both forms of IDH normally facilitate the reversible oxidative decarboxylation of isocitrate to α-ketoglutarate in the tricarboxylic acid cycle [75]. This reaction reduces nicotinamide adenine dinucleotide phosphate (NADP+) to NADPH, which is an essential cofactor for many biochemical processes, and its homeostasis plays a role in many cancers [76]. Mutations in *IDH1/2* are gain-of-function mutations that instead convert its normal product, α-ketoglutarate, to 2-hydroxyglutarate (2-HG), an oncometabolite that consumes NADPH (Figure 1a) [77]. Additionally, 2-HG leads to mechanisms that trigger apoptosis, in which anti-apoptotic B-cell lymphoma protein 2 (BCL-2) prevents apoptosis [78]. Consequently, *IDH* mutant cells become dependent on BCL-2, making them more susceptible to BCL-2 inhibition, which is grounds for a therapy regimen we will mention later in this review. The enzyme ten eleven translocation 2 (TET2) catalyzes the reaction to create the active product for the demethylation of cytosine [79]. Mutations in TET2 and *IDH1/2* are mutually exclusive in AML patients, and loss-of-function mutations in TET2 have outcomes similar to those of *IDH1/2* mutants [80]. TET2 is dependent on α-ketoglutarate, to which 2-HG, the structural analog and mutant IDH oncometabolite, binds and competitively inhibits TET2 function [81]. Consequently, 2-HG, specifically the R-enantiomer, disrupts cellular differentiation through the inactivation of chromatin by DNA and histone hypermethylation of the hematopoietic stem cell genome, contributing to leukemogenesis [80,82]. Mutations in *IDH* have been detected before overt leukemia and especially in the presence of additional driver mutations, likely rendering hematopoietic stem cells more susceptible to leukemic transformation [83].

### 5.2. The Controversial Prognostic Impact of IDH1/2 Mutations in AML

We have discussed examples of driver mutations in AML that contribute to favorable, intermediate, and adverse risk. However, we now discuss the controversial prognostic significance of *IDH1/2* mutations, despite their high prevalence in AML [84]. There have been various studies investigating the prognostic impact of *IDH*, and some demonstrate that it may be dependent on the location of the mutation and the presence of the *FLT3*-ITD genotype [85,86]. In a prospective analysis of AML patients with *IDH* mutations, it has been shown that those with increased levels of 2-HG during complete remission had shorter overall survival than patients with lower levels of 2-HG (*p* = 0.02) [87]. Patients with *IDH* mutations also had an increased incidence of *NPM1* and *FLT3*-ITD mutations, intermediate risk cytogenetics, and increased bone marrow blasts at diagnosis [88]. However, co-occurring *IDH* mutations do not override the favorable prognosis of *NPM1* mutations in patients younger than 60 years old. Additionally, patients without co-occurring *IDH*, *NPM1*, *FLT3*-ITD, or *DNMT3A* mutations had inferior outcomes [89]. Mutations at R132 in *IDH1* and R172/R140 in *IDH2* are associated with increased levels of 2-HG in AML patients [77]. Additionally, the prognostic impact of the location of the mutation is inconsistent, with some studies showing that *IDH2* mutations at R140 have a more favorable prognosis than *IDH1* mutations at R132, and other studies finding no significant difference [20,86]. However, *IDH2* R140 mutations frequently cooccur with *NPM1* mutations, which is associated with a favorable prognosis in newly diagnosed AML but a poor prognosis in relapsed/refractory AML [89,90]. The association with *NPM1* may explain the favorable prognosis with R140 *IDH2* mutations. The unclear prognostic significance of *IDH* mutations may be due to the vast heterogeneity of co-occurring mutations and the various locations where they may occur, making genetic profiling and translational research essential in clarifying the role of *IDH* in AML.

### 5.3. Targeting IDH Mutations in AML: Efficacy of IDH Inhibitors and Combination Therapies

*IDH1* mutations have an extra level of complexity compared to *IDH2* mutations, as they are more prone to resistance [91]. Due to the recent discovery of the role of *IDH* mutations in the survival outcomes of AML, targeted therapies have been developed to inhibit the activity of *IDH* mutated enzymes. *IDH* inhibitors bind to the active site of the IDH enzyme and prevent the release of 2-HG, restoring α-ketoglutarate levels and cellular differentiation to normal. Differentiation syndrome is a common adverse effect occurring in about 19% of patients treated with *IDH* inhibitors and can lead to tissue damage and inflammation due to the proliferation of differentiated leukemic cells that alter cytokine levels [92,93].

Ivosidenib was first FDA-approved in 2018 for relapsed/refractory AML patients (NCT02074839) [94]. Following this, a phase III trial (NCT03173248) showed that the combination of ivosidenib and azacitidine was effective in newly diagnosed AML patients with *IDH1* mutations. As of 2022, it is FDA-approved as a first-line therapy due to the AGILE study showing a median overall survival of 24 months with ivosidenib and azacitidine and 7.9 months with placebo and azacitidine, (*p* = 0.001) [95]. However, it should be noted that this study had limitations, including the control arm being sub-optimal care at the time and the primary endpoint being changed [96]. A newer *IDH1* inhibitor FDA-approved in 2022, olutasidenib, has shown longer durations of complete remission in a phase I/II study (NCT02719574) with *IDH1* mutant AML patients compared to ivosidenib, although it has not yet been investigated in a head-to-head trial [94]. In 153 relapsed/refractory AML patients receiving olutasidenib as monotherapy, the median duration of complete remission was 28.1 months, and the rate of complete remission was 32% [97].

As for *IDH2* mutations, enasidenib was FDA-approved in 2017 for relapsed/refractory AML patients, and a phase III trial (NCT02577406) comparing enasidenib to conventional care regimens respectively showed improved event-free survival (4.9 vs. 2.6 months) and overall response rate (40.5% vs. 9.9%), although the study did not indicate improved overall survival [98].

Due to the susceptibility of BCL-2 inhibition in *IDH*-mutant AML patients mentioned previously, the combination of *IDH* inhibitors with venetoclax, a BCL-2 inhibitor, is being explored. The phase Ib/II VIALE-A study (NCT03471260) evaluated the use of ivosidenib and venetoclax with or without azacitidine as frontline therapy in relapsed/refractory patients and showed that it was safe in *IDH1*-mutated myeloid malignancies and appeared to escape mechanisms of resistance that have been observed with the use of single-agent *IDH* inhibitors [99]. This study in the phase III setting may show the true use of ivosidenib in frontline vs. relapsed/refractory patients with *IDH* mutations, due to the limitations of past trials in the frontline setting.

## 6. Discussion

The role of driver mutations is critical in the pathogenesis and treatment of AML. For an overview of AML driver mutations and therapy strategies, see Table 1. Prognosis, treatment response, and overall patient outcomes are influenced by these genetic alterations, but each patient is unique due to the heterogeneity of co-occurring mutations in hematopoietic stem cells. Clinical trials have been successful in proving the efficacy of targeted therapies for *FLT3*-ITD and *IDH1/2*, but finding a targeted agent for *NPM1* and *TP53* remains a challenge.

## 7. Conclusions

There is a profound implication for driver mutations in the treatment of AML, and advances in diagnostic assays for measuring residual disease are a topic of ongoing research, in addition to exploring novel therapeutic agents. As our understanding of the molecular underpinnings of AML deepens, the potential for precision medicine increases and offers hope for less toxic and more effective therapies.

## Figures and Tables

**Table 1:** Overview of AML Driver Mutations and Therapy Strategies.

Driver Gene Mutation	Biological Consequence	Frequency in AML (%)	Associated Clinical Features	Current FDA Approved Treatments for AML	Emerging Therapies	References
*FLT3-ITD*	Constitutive activation of the tyrosine kinase receptor, leading to the potential for uncontrolled malignant proliferation of hematopoietic cells.	20–30%	intermediate risk (ELN 2022)	TN ^1^: midostaurin + chemotherapy (NCT00651261) TN: quizartinib + chemotherapy (NCT02039726) R/R: gilteritinib (NCT02421939)	crenolanib (NCT03258931)	[1,3,7,48,50,52,54]
*NPM1*	Impairs the p53 tumor suppressor pathway, reducing apoptosis, and disrupts the nuclear export of ribosomal proteins, allowing myeloid cells to proliferate.	30%	favorable prognosis in the absence of FLT3-ITD mutations (ELN 2022)	TN: venetoclax + hypomethylating agent (NCT02993523) TN: chemotherapy	R/R: ziftomenib (NCT04067336) R/R: revumenib (NCT04065399)	[3,100,101]
*TP53*	Failure to maintain genomic stability, regulate the cell cycle, and induce apoptosis, which drives leukemogenesis by allowing the survival and proliferation of genetically unstable cells.	10%	adverse risk (ELN 2022)	TN: venetoclax + hypomethylating agent (NCT02993523) TN: chemotherapy	Combination therapies:	[3,101]
*IDH1/2*	Disruption of cellular differentiation through the inactivation of chromatin by DNA and histone hypermethylation, contributing to leukemogenesis.	*IDH1*: 5–10% *IDH2*: 15–20%	Prognostic significance is controversial, although associated with decreased survival.	*IDH1*: TN: ivosidenib + azacitidine (NCT03173248) R/R: olutasidenib (NCT02719574) *IDH2*: R/R: enasidenib (NCT02577406)	Combination therapies: ivosidenib with venetoclax ± azacitidine (NCT03471260)	[3,80,82,95,97,98]

^1^ Abbreviations: TN: Treatment Naïve; R/R: Relapsed/Refractory.

## Data Availability

Not applicable.
